# Prediction of false-positive PI-RADS 5 lesions on prostate multiparametric MRI: development and internal validation of a clinical-radiological characteristics based nomogram

**DOI:** 10.1186/s12894-024-01465-0

**Published:** 2024-04-02

**Authors:** Yongbing Cheng, Bo Fan, Yao Fu, Haoli Yin, Jiaming Lu, Danyan Li, Xiaogong Li, Xuefeng Qiu, Hongqian Guo

**Affiliations:** 1 Department of Urology, Nanjing Drum Tower Hospital, Affiliated Hospital of Medical School, Nanjing University, Nanjing, 210008 China; 2 Institute of Urology, Nanjing University, Nanjing, China; 3 Department of Urology, The First People’s Hospital of Changshu, The Changshu Hospital Affiliated to Soochow University, Changshu, China; 4 Department of Pathology, Nanjing Drum Tower Hospital, Affiliated Hospital of Medical School, Nanjing University, Nanjing, China; 5 Department of Radiology, Nanjing Drum Tower Hospital, Affiliated Hospital of Medical School, Nanjing University, Nanjing, China

**Keywords:** Prostate cancer, Biopsy, Magnetic resonance imaging, PI-RADS 5, Nomogram

## Abstract

**Background:**

To develop a risk model including clinical and radiological characteristics to predict false-positive The Prostate Imaging Reporting and Data System (PI-RADS) 5 lesions.

**Methods:**

Data of 612 biopsy-naïve patients who had undergone multiparametric magnetic resonance imaging (mpMRI) before prostate biopsy were collected. Clinical variables and radiological variables on mpMRI were adopted. Lesions were divided into the training and validation cohort randomly. Stepwise multivariate logistic regression analysis with backward elimination was performed to screen out variables with significant difference. A diagnostic nomogram was developed in the training cohort and further validated in the validation cohort. Calibration curve and receiver operating characteristic (ROC) analysis were also performed.

**Results:**

296 PI-RADS 5 lesions in 294 patients were randomly divided into the training and validation cohort (208 : 88). 132 and 56 lesions were confirmed to be clinically significant prostate cancer in the training and validation cohort respectively. The diagnostic nomogram was developed based on prostate specific antigen density, the maximum diameter of lesion, zonality of lesion, apparent diffusion coefficient minimum value and apparent diffusion coefficient minimum value ratio. The C-index of the model was 0.821 in the training cohort and 0.871 in the validation cohort. The calibration curve showed good agreement between the estimation and observation in the two cohorts. When the optimal cutoff values of ROC were 0.288 in the validation cohort, the sensitivity, specificity, PPV, and NPV were 90.6%, 67.9%, 61.7%, and 92.7% in the validation cohort, potentially avoiding 9.7% unnecessary prostate biopsies.

**Conclusions:**

We developed and validated a diagnostic nomogram by including 5 factors. False positive PI-RADS 5 lesions could be distinguished from clinically significant ones, thus avoiding unnecessary prostate biopsy.

**Supplementary Information:**

The online version contains supplementary material available at 10.1186/s12894-024-01465-0.

## Background

Prostate cancer (PCa) is the second solid tumor and the fifth reason for cancer-related death in men worldwidely [1]. Multiparametric magnetic resonance imaging (mpMRI) is currently the standard imaging of primary prostate cancer due to its good sensitivity and specificity for the detection and localization of clinically significant PCa (csPCa) lesions [2]. The Prostate Imaging Reporting and Data System (PI-RADS), which has been updated to version 2.1, is a standardized reporting system to assess prostate malignant-suspected lesions based on the information from mpMRI [3]. A 5-grade scale was used to describe the likelihood of suspicious PCa lesions detected on mpMRI.

With the advances in prostate mpMRI, missed detection of csPCa has been significantly decreased [4]. Patients with positive lesions (PI-RADS ≥ 3) on mpMRI were recommended for prostate biopsy [5]. However, PI-RADS system has some limitations caused by imaging mimics and pitfalls that can be misinterpreted as PCa [6], leading to an inevitable false positive. The inter-reader variability was also a limitation of mpMRI [7]. Therefore, false-positive lesions are a new problem in the era of MRI, which prompts unnecessary prostate biopsies. Lesions with a PI-RADS score of 5 (PI-RADS 5 lesions) on prostate mpMRI have been reported to be highly correlated with csPCa, with the average positive predictive value (PPV) of 72% [8]. A prostate biopsy would be strongly recommended to men with PI-RADS 5 lesions. Of note, 12.7–15.5% of negative biopsy rate and 5.3–18.7% of detection rate of clinically insignificant PCa were reported in PI-RADS 5 lesions [9, 10]. Therefore, a preselection is still needed to avoid unnecessary biopsy-related stress and complications [11] in men with PI-RADS 5 lesions. Few studies have been conducted to evaluate the risk factors or models to predict false-positive PI-RADS 5 lesions [10–12]. However, the results are far from being widely adopted in clinical practice due to the small sample size, limited included parameters, and lack of validation.

This retrospective study was designed to develop a risk model by including clinical and radiological characteristics to predict false-positive PI-RADS 5 lesions (benign or clinically insignificant prostate cancer lesions), using MRI/Ultrasound fusion targeted biopsy as the reference. In addition, an internal validation was performed to assess the efficacy of our developed risk model.

## Patients and methods

### Patients

Between September 2018 and November 2021, data of 612 biopsy-naive patients who had undergone mpMRI before prostate biopsy in our center were retrospectively collected. The study was approved by the Institutional Review Board of Nanjing Drum Tower Hospital and compliant with a waiver of the requirement for informed consent.

The inclusion criteria were: [1] PI-RADS 5 lesions were detected on mpMRI in one month before biopsy; [2] MRI/ultrasound fusion targeted biopsy (TB) was performed for the suspicious lesions on mpMRI; [3] Prostate specific antigen (PSA) results in one week before biopsy. Patients who had previous prostate-related treatments (e.g. surgery, radiology, chemical and hormone treatment) or other tumor histories, and whose MRI images were not clear or had artifacts were excluded. The patient selection flowchart is shown in Supplementary Fig. 1.

### MRI examination paraments

All prostate mpMRI data were obtained from a 3.0T MRI scanner (Achieva 3.0 T TX, Philips Medical Systems, the Netherlands) with a 32-channel body coil (In vivo) according to the previously described protocol [13] No endorectal coil was used. All data for each patient included transverse, coronal and sagittal T2-weighted (18 slices, thickness 3 mm/gap 0.5 mm, TR 3744 ms, TE 120 ms, number of signals acquired 2, resolution 1.49 mm × 1.51 mm) turbo spin-echo images. Diffusion weighted imaging (DWI), spin-echo echo-planar images (18 slices, thickness 3 mm, intersection gap 1 mm, TR 925/TE 41 ms, number of signals acquired 1, resolution 3 mm × 3 mm, b-factor 0/800/1500 s/mm^2^) were also obtained. The apparent diffusion coefficient (ADC) map was generated with the United Imaging software from the DWI data on a Philips workstation.

All images were respectively read by 2 board-certified, subspecialized abdominal radiologists, who had 5 years of MRI prostate diagnosis experience and master the PI-RADS V2 scoring system [14]. Lesions were evaluated respectively on the base of the scoring system.

### Prostate biopsy and histopathologic evaluation

PI-RADS 5 index lesions, as target lesions, were contoured by radiologists using special imaging software (Philips workstation). TB was performed using MRI/Ultrasound fusion technique with a transperineal ultrasound-guided biopsy system [15]. Biopsies were performed by one urologist with over 5 years of experience in prostate biopsy. Each target lesion had 2 biopsy cores, and 12-core systematic biopsy was performed after TB with a template map that was generated by the fusion software with lesion location hidden.

Histopathologic evaluation was conducted by a dedicated genitourinary pathologist with over 10 years of experience. Gleason grading was performed according to 2014 International Society of Urological Pathology guidelines [16].

### Clinical and radiological variables

For each patient with PI-RADS 5 lesions, demographic, clinical, MRI, and pathologic data were collected. Demographic data included age, and clinical data included PSA level. MRI data included prostate volume, the maximum diameter of lesion, area of lesion, zonality of lesion, apparent diffusion coefficient minimum value (ADCmin), apparent diffusion coefficient mean value (ADCmean). The prostate volume was calculated by the formula: [maximum anteroposterior diameter] × [maximum transverse diameter] × [maximum longitudinal diameter] × 0.52. The maximum diameter and area of lesion were measured in the largest dimension in the axial plane and lesion in peripheral zone (PZ) and transition zone (TZ) was measured on DWI and T2-weighted image respectively. Zonality of lesion was divided into PZ, TZ, and PZ + TZ. Region of interest (ROI) was extracted in the largest dimension, encompassing the darkest lesion area on the axial image from the ADC map. The contrast region was also drawn in the normal zone corresponding with ROI in the same dimension. ADCmean and ADCmin of ROI were calculated by the special software (Philips workstation). Apparent diffusion coefficient minimum value ratio (ADCminr) and apparent diffusion coefficient mean value ratio (ADCmeanr) were defined as the ratio between values in ROI and contrast region. Prostate specific antigen density (PSAD) was calculated by dividing PSA by prostate volume. Pathologic data included the histopathologic results of targeted biopsy of the index PI-RADS 5 lesion.

### Statistical analysis

Continuous variables were presented as median (interquartile range, IQR) and compared using an independent sample test. Categorical variables were expressed as frequency (proportion), and the chi-square test or Fisher exact test were adopted for comparisons. For categorical variables, dummy variables were set in multivariate logistic regression analysis. A nomogram was performed to distinguish false-positive lesions based on the stepwise multivariate analysis with backward elimination, using the *rms* package of R. The performance of the nomogram was quantified by concordance index (C index) and calibration with 1000 bootstrap samples to decrease the overfit bias for the training and validation cohort. Receiver operating characteristic (ROC) analysis was employed to calculate the optimal cutoff value that was determined by maximizing the Youden index (i.e., sensitivity + specificity-1). Accuracy of the optimal cutoff value was assessed by the sensitivity, specificity, predictive values, and likelihood ratios. Decision curve analysis (DCA) was performed to evaluate the clinical usefulness of the model. All analyses were performed using R, version 4.2.0 (http://www.r-project.org/) with a 2-tailed statistical significance level set at *P* < 0.05.

## Results

### Patient characteristics

296 lesions in 294 patients were analyzed in our study. All lesions were randomly divided into the training cohort (208 lesions) and validation cohort (88 lesions) respectively. Of 208 training cohort lesions, 132 (63.5%) were histopathologically confirmed as clinically significant lesions while 76 (36.5%) were benign or clinically insignificant. Of 88 validation cohort lesions, 56(63.6%) were confirmed to be clinically significant while 32(36.4%) were benign or clinically insignificant. Detailed information about patients’ characteristics was shown in Table 1. There was a significant difference between the two cohorts in variables: Zonality of lesion, ADCmin, ADCmeanr. Of note, the pathologic features of false-positive lesions were analyzed through biopsy slices and 28 (50%) benign lesions were histopathologically confirmed as chronic inflammatory, also called chronic prostatitis (Supplementary Table 1).

**Table 1:** Participants characteristics

	Cohort, No. (%)		
Variable	Training (*n* = 208)	Validation (*n* = 88)	*P* Value
Median age, yr (IQR)	72.0 (12.0)	72.0 (11.2)	0.917
Median PSA, ng/ml (IQR)	12.0 (13.5)	10.1 (11.7)	0.844
Median prostate volume, cm^3^, (IQR)	39.5 (26.4)	37.0 (28.3)	0.497
Median PSAD, ng/ml/cm^3^, (IQR)	0.3 (0.4)	0.3 (0.3)	0.171
The median maximum diameter of lesion, cm (IQR)	19.6 (8.2)	19.5 (5.7)	0.059
Median area of lesion, mm^2^ (IQR)	232.0 (162.2)	251.0 (154.8)	0.674
Zonality of lesion, n(%)			
Peripheral zone	94 (45.2)	34 (38.6)	0.040
Transition zone	96 (46.2)	52 (59.1)	
Peripheral zone and transition zone	18 (8.7)	2 (2.3)	
Outcome			
Clinically significant prostate cancer	132 (63.5)	56 (63.6)	1.000
Benign and clinically insignificant prostate cancer	76 (36.5)	32 (36.4)	
ISUP Grade, n(%)			
0 (benign)	40(21.6)	16(20.5)	0.756
1	36(15.9)	16(17.0)	
2	49(22.6)	25(27.3)	
3	41(19.7)	18(20.5)	
4	37(17.8)	10(11.4)	
5	5(2.4)	3(3.4)	
Median ADCmin (IQR)	433.5 (252.8)	448.5 (200.5)	0.224
Median ADCmean (IQR)	661.0 (250.8)	669.0 (234.0)	0.792
Median ADCminr (IQR)	0.5 (0.3)	0.5 (0.2)	0.556
Median ADCmeanr (IQR)	0.5 (0.2)	0.5 (0.2)	0.077

IQR: interquartile range; PSA: prostate-specific antigen; PSAD: prostate-specific antigen density; ISUP: International Society of Urological Pathology; ADCmin: apparent diffusion coefficient minimum value; ADCmean: apparent diffusion coefficient mean value; ADCminr: apparent diffusion coefficient minimum value ratio; ADCmeanr: apparent diffusion coefficient mean value ratio

### Variables selection

The variables were screen out by stepwise method with backward elimination. Finally, PSAD, the maximum diameter of lesion, zonality of lesion, ADCmin and ADCminr, which were shown to be significant in the multivariable analysis, were selected as independent predictors for the false-positive lesions in our risk model (Table 2).

**Table 2:** Multivariate logistic regression analysis to predict false-positive lesions in the training cohort

Variable	β	OR (95% CI)	P Value
PSAD, ng/ml/cm^3^	-1.612	0.200 (0.069–0.477)	< 0.001
The median maximum diameter of lesion, mm	0.100	1.105 (1.043–1.178)	0.001
Zonality of lesion			
Peripheral zone	Reference		
Transition zone	0.789	2.200 (1.065–4.633)	0.035
Peripheral zone and transition zone	2.692	14.756(3.545–71.612)	< 0.001
ADCmin	0.003	1.003 (1.001–1.006)	0.018
ADCminr	2.270	9.675 (1.478–89.009)	0.037

OR: odds ratio; CI: confidence interval; PSAD: prostate-specific antigen density; ADCmin: apparent diffusion coefficient minimum value; ADCmean: apparent diffusion coefficient mean value; ADCminr: apparent diffusion coefficient min value ratio

### Development and validation of nomogram

**Fig. 1:**
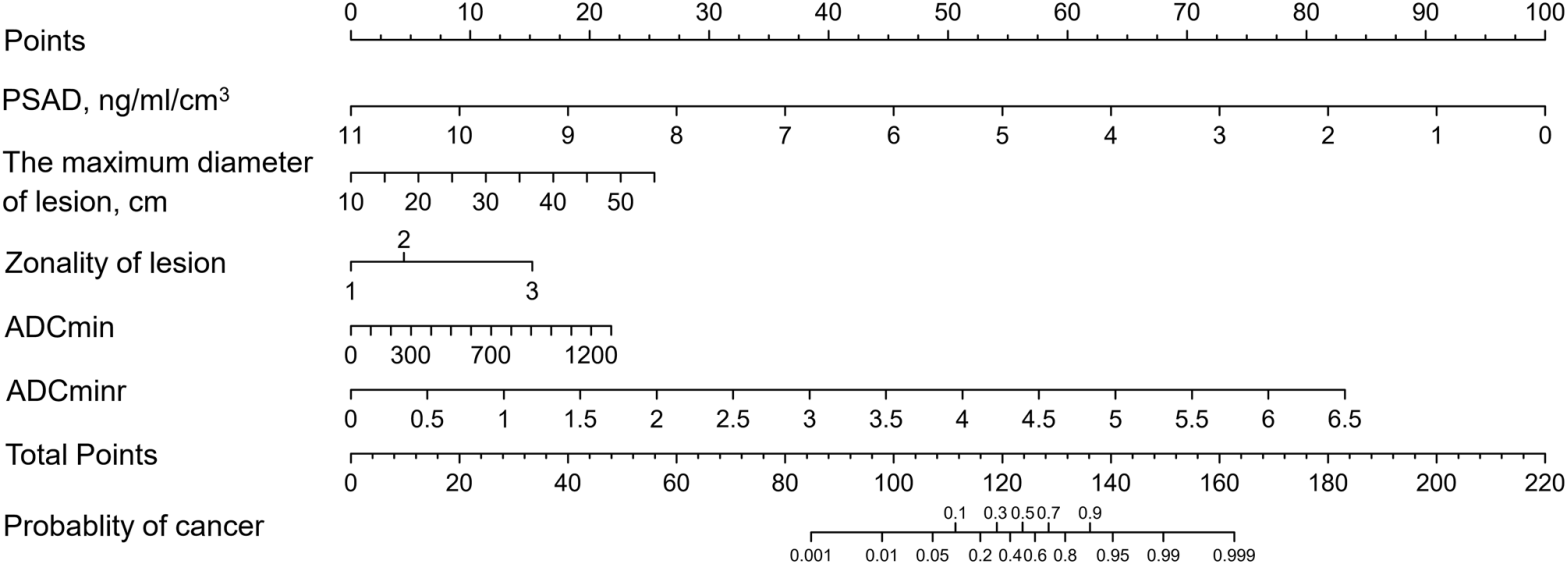
Nomogram for predicting false-positive PI-RADS 5 lesions. PSAD: prostate specific antigen density; ADCmin: apparent diffusion coefficient minimum value; ADCminr: apparent diffusion coefficient minimum value ratio

**Fig. 2:**
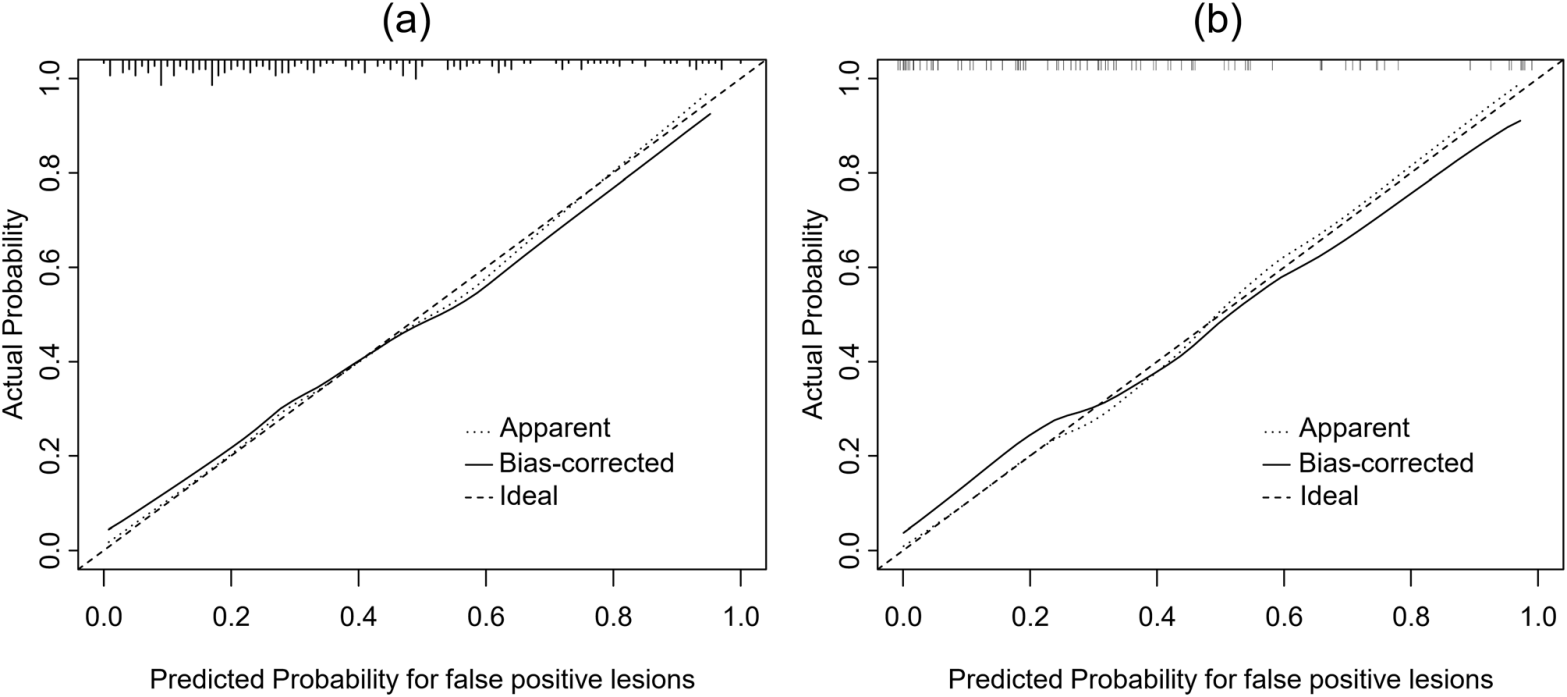
Calibration curves for prediction of false-positive PI-RADS 5 lesions in the training cohort (**a**) and validation cohort (**b**)

A false-positive lesion risk estimation nomogram was built based on the multivariate logistic regression model and was validated using the bootstrap validation method (Fig. 1). The C index of the model in the training cohort is 0.821, which showed good accuracy in estimating the risk of false-positive lesions. Furthermore, the good agreement on the presence of false-positive lesions between the risk estimation by the nomogram and histopathologic confirmation on target biopsy specimens was reflected from the calibration curve graphically (mean absolute error 0.022; Fig. 2a). The nomogram showed a C index of 0.871 in the validation cohort and the calibration curve was good (mean absolute error 0.031; Fig. 2b).

### Risk of malignancy on the nomogram scores

**Fig. 3:**
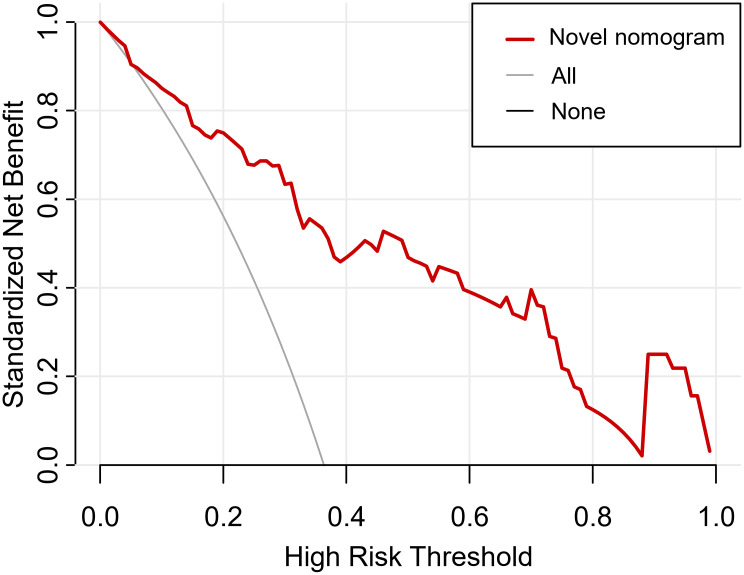
Decision curve analysis for the nomogram

The optimal cutoff values of the ROC curves, in the training cohort and validation cohort respectively, were estimated to be 0.290 and 0.288. The sensitivity, specificity, positive predictive value, and negative predictive value in differentiating the presence of false-positive PI-RADS 5 lesions were 81.6%, 68.9%, 60.2%, and 86.7% in the training cohort, and 90.6%, 67.9%, 61.7%, and 92.7% in the validation cohort, respectively (Table 3). The DCA revealed that the use of the nomogram has more net benefits (Fig. 3).

**Table 3:** Accuracy of the prediction score of the nomogram for estimating the risk of false-positive lesions

	Value (95% CI)	
Variable	Training Cohort	Validation Cohort
Lesion area under ROC curve, concordance index	0.821 (0.762–0.880)	0.871 (0.794–0.947)
Cutoff value	0.290	0.288
Sensitivity, %	81.6 (72.9–90.3)	90.6 (80.5–100)
Specificity, %	68.9 (61.0-76.8)	67.9 (55.6–80.1)
Positive predictive value, %	60.2 (50.7–69.6)	61.7 (47.8–75.6)
Negative predictive value, %	86.7 (80.2–93.2)	92.7 (84.7–100)
Positive likelihood ratio	2.626 (1.994–3.460)	2.819 (1.896–4.192)
Negative likelihood ratio	0.867 (0.802–0.932)	0.138 (0.046–0.412)

ROC: receiver operating characteristic

## Discussion

The false-positive of PI-RADS 5 lesions would cause unnecessary prostate biopsies. In the present study, using the MRI-guided DWI as the standard reference, we developed a diagnostic nomogram with good performance in predicting the false-positive of PI-RADS 5 lesions on prostate mpMRI. In addition, the internal validation using a validation cohort further confirmed the accuracy of our developed risk model with a C-index of 0.871. Our developed nomogram showed good performance in differentiating benign and clinically insignificant PI-RADS 5 lesions from clinically significant ones, according to the validation cohort. To the best of our knowledge, this was the first study to develop a nomogram to predict false-positive PI-RADS 5 lesions with internal validation.

Prostate mpMRI, which incorporates anatomic and functional techniques in a multiparametric approach, is currently the standard imaging for the diagnosis of csPCa [17]. In addition, prostate mpMRI has been increasingly applied in local staging [18], nerve-sparing technique [19], focal therapy [20], and post-treatment follow-up [21]. However, it has been reported that a range of normal anatomic structures (e.g. anterior fibromuscular stroma, central zone, surgical capsule, periprostatic vein, periprostatic lymph nodes) and benign diseases (e.g. benign prostatic hyperplasia, prostatitis) mimicked PCa on mpMRI [22], leading to potential pitfalls in imaging interpretation. Taking PI-RADS 5 lesions for example, Sandra et al [11] reported that the false-positive rate of TB with PI-RADS 5 lesions was 13.1% for all PCa, and 27.6% for csPCa respectively. Recently, Davenport et al [23] reported that in 381 patients with PI-RADS 5 lesions, 76 patients (19.9%) presented negative lesions in biopsy results. In the present study, around 36.5% PI-RADS 5 lesions were finally confirmed as false-positive using the TB-derived histopathology as the reference. Thus, it is clinically important to analyze the clinical and radiographic features of false-positive PI-RADS 5 lesions and differentiate them from clinically significant prostate cancer lesions, avoiding up to 36.5% unnecessary biopsy.

Few retrospective studies have been designed to analyze the clinical and radiological features of the false-positive PI-RADS 5 lesions. Sheridan et al. analyzed 98 PI-RADS 5 lesions identified in 89 patients [8]. The authors found that lower PSAD and apex or base location were significantly associated with false-positive lesions. Polanec [12] investigated 101 PI-RADS 4 or 5 lesions in 101 men. By comparing quantitative ADC value derived from DWI measurement, they found significantly lower ADC value in malignant lesions, suggesting the potential role of quantitative ADC value in avoiding up to 33% unnecessary MRI-guided TB [12]. Similarly, Apfelbeck [6] found transitional zone lesions, prostate volume and pre-biopsy-status were found to be correlated with false-positive PI-RADS 4 or 5 lesions on mpMRI [6]. Compare to the previously published studies, our studies focused on PI-RADS 5 lesions with a much larger sample size. In addition, we included easily available clinical and radiological variables for multivariate logistic regression analyses. An easy-to-use nomogram with good performance was developed by incorporating 5 comprehensive variables. Internal validation was performed to further validate the performance of our developed risk model.

In our risk model, lower PSAD and higher ADC values were associated with false-positive lesions, which was consistent with the previously published studies [8, 11, 12, 24]. Of interest, our study found that a larger lesion diameter was significantly associated with benign lesions, which was opposite to the results in Stavrinides et al.’s study indicating that smaller index lesions were associated with “false-positive” lesions [11]. However, “false-positive” lesions were defined as suspicious lesions (Likert score 3–5 on mpMRI) but no/insignificant cancer on biopsy results. In addition, histopathology derived from transperineal mapping biopsy, instead of MRI-guided TB, was used as the reference. As shown in the Supplementary Table 1, chronic prostatitis, which has been well described to have significantly diffuse morphology on MRI [25, 26], was detected in 55% false-positive PI-RADS 5 lesions. This might explain why false-positive lesions in the present study are associated with larger lesion diameter. PSAD could contribute to distinguish the inflammation lesions from prostate cancer [27].

There were some limitations in this study. First was the retrospective nature of this single-center study. A prospective multi-center study with external validation would be necessary to further confirm the reliability of our developed risk model. However, an internal validation was performed in our study to confirm the performance of our developed model. Second, MRI/Ultrasound fusion TB-derived histopathological results were set as the standard reference. However, TB could not localize some specific lesions perfectly, potentially leading to the misdiagnosis of suspicious lesions. Two biopsy cores were set for each lesion to decrease the inaccuracy. Third, the ROI was drawn manually, which might affect the stability and repeatability of the model. Two experienced radiologists draw the ROI blindly to avoid potential bias.

## Conclusions

We developed and internally validated an easy-to-use diagnostic nomogram by including 5 clinical and radiological variables. Using this model, false-positive lesions could be distinguished from clinically significant ones, potentially avoiding up to 9.7% unnecessary prostate biopsy in men with PI-RADS 5 lesions on mpMRI, according to the validation cohort.

### Electronic supplementary material

Below is the link to the electronic supplementary material.

Supplementary Material 1

Supplementary Material 2

## Data Availability

The datasets used and analyzed during the current study are available from the corresponding author on reasonable request.
