# Supplementary figures and images for: Prediction of false-positive PI-RADS 5 lesions on prostate multiparametric MRI: development and internal validation of a clinical-radiological characteristics based nomogram
Source: BMC Urol. 2024 Apr 2;24:76. doi: 10.1186/s12894-024-01465-0 (PMC10986137; doi:10.1186/s12894-024-01465-0)

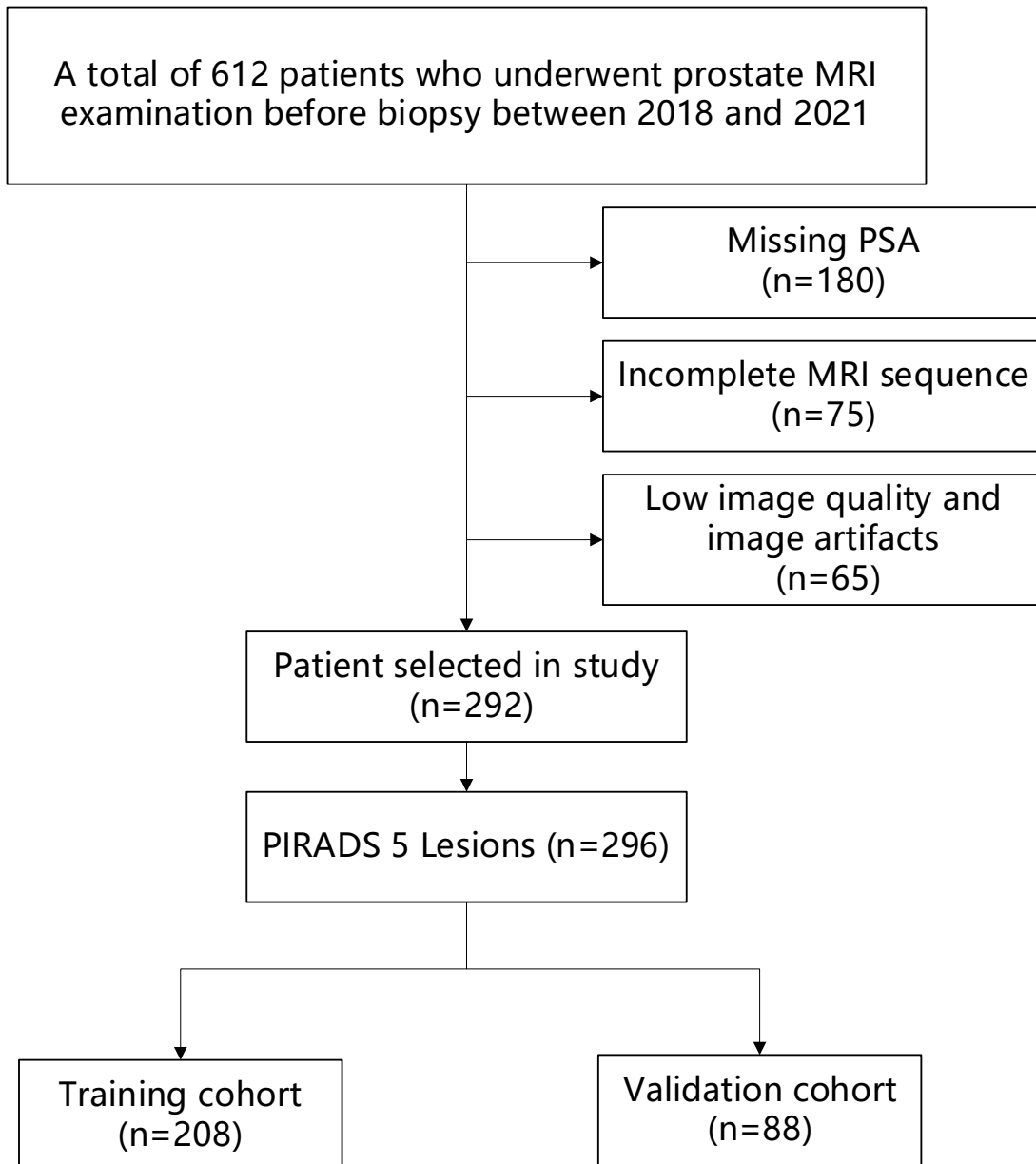

Supplement: Supplementary file 1 — Supplementary Material 1 [file 12894_2024_1465_MOESM1_ESM.pdf]
