# Supplementary material for: Prediction of false-positive PI-RADS 5 lesions on prostate multiparametric MRI: development and internal validation of a clinical-radiological characteristics based nomogram
Source: BMC Urol. 2024 Apr 2;24:76. doi: 10.1186/s12894-024-01465-0 (PMC10986137; doi:10.1186/s12894-024-01465-0)
Supplement: Supplementary file 2 — Supplementary Material 2 [file 12894_2024_1465_MOESM2_ESM.docx]

Supplementary Table 1 Pathologic features of false positive lesions

| Pathologic features | No. (%) |
| --- | --- |
| Clinically insignificant prostate cancer | 52 |
| Benign | 56 |
| Stromal tumor of uncertain malignant potential (STUMP) | 1 (1.7) |
| Nonspecific granulomatous prostatitis (NSGP) | 3 (5.3) |
| Stroma hyperplasia | 19 (33.9) |
| Chorionic inflammatory | 28 (50.0) |
| Gland hyperplasia | 2 (3.6) |
| Normal tissue | 3 (5.3) |
